# Morphological Analyses of W/Cu Functional Graded Materials Obtained by Conventional and Spark Plasma Sintering

**DOI:** 10.3390/ma16114126

**Published:** 2023-06-01

**Authors:** Claudiu Nicolicescu, Victor Horia Nicoară, Cristina Ileana Pascu, Ștefan Gheorghe, Cristian Oliviu Burada, Traian Florin Marinca, Florin Popa

**Affiliations:** 1 Faculty of Mechanics, University of Craiova, 200512 Craiova, Romania; victor.nicoara@edu.ucv.ro (V.H.N.); stefan.gheorghe@edu.ucv.ro (Ș.G.); cristian.burada@edu.ucv.ro (C.O.B.); 2 Faculty of Materials Engineering and Environment, Technical University of Cluj-Napoca, 400641 Cluj-Napoca, Romania; traian.marinca@stm.utcluj.ro (T.F.M.); florin.popa@stm.utcluj.ro (F.P.)

**Keywords:** spark plasma sintering, conventional sintering, functional graded materials, scanning electron microscopy

## Abstract

The paper presents the analysis of two compaction methods for obtaining W/Cu Functional Graded Materials (FGMs) consisting of three layers with the following compositions (% weight): first layer 80 W/20 Cu, second layer 75 W/25 Cu, and third layer 65 W/35 Cu. Each layer composition was obtained using powders obtained through mechanical milling. The two compaction methods were Spark Plasma Sintering (SPS) and Conventional Sintering (CS). The samples obtained after the SPS and CS were investigated from morphological (scanning electron microscopy-SEM) and compositional (energy dispersive X-ray spectroscopy-EDX) points of views. Additionally, the porosities and the densities of each layer in both cases were studied. It was found that the densities of the sample’s layers obtained through SPS are superior to those obtained through CS. The research emphasizes that, from a morphological point of view, the SPS process is recommended for W/Cu-FGMs, having raw materials as fine-graded powders against the CS process.

## 1. Introduction

Materials, energy, and modern sciences are the three great pillars of modern technology. In recent years, materials science has developed rapidly due to interdisciplinarity and due to new theories and technologies. The development of materials that respond to practical applications is of great interest, especially if their manufacturing technologies ensure low energy consumption and environmental protection [1].

Materials based on W/Cu are widely used for electrical contacts, heat sink materials for high-power microelectronic devices, welding electrodes, and thermal management devices [2,3]. Due to the lack of solubility between tungsten (W) and copper (Cu), these kinds of materials are difficult to produce.

There are several methods to produce W/Cu materials: by infiltrating the liquid copper into open pores of W skeleton and by liquid or solid phase sintering. The infiltration method requires higher consumption of energy, especially for complex shape parts, so it is very expensive [1,4]. An alternative for mixing W with Cu is mechanical milling. This approach was investigated in research [5] that showed the potential of the method.

Additionally, in ref. [6], research about obtaining W-Cu pseudo-alloys produced via powder injection molding (PIM) with powders prepared thermochemically is presented. By applying this treatment, the increase of the values of physical-mechanical properties of the final pieces was observed. In ref. [7], a novel approach to electric explosion of intertwined wires based on W-Cu powders and extrusion 3D printing was applied.

Besides a simple W/Cu mix, an improvement of this material class is obtaining functional graded materials. FGMs represent a class of materials composed of one or more different materials whose properties and structure vary throughout their volume. The component materials can vary chemically from metallic, ceramic, and polymeric materials to inorganic/organic materials, such as cells and tissues. A concrete example of biological materials with a structural and functional gradient is bones, teeth, and bamboo wood [8].

From a geometric point of view, the materials that make up an FGM can be in the form of lamella, granules, fibers, etc. [9].

Due to their high performance and many of their functionalities, FGMs have been successfully used in the construction of new generations of spacecraft. The most familiar FGMs are those composed of refractory ceramic materials and metals because they combine properties very well, such as resistance to high temperatures and wear and oxidation offered by ceramic materials with properties such as mechanical strength, machining, and toughness offered by metallic materials [10]. Due to their graded transition, another very important field of application of FGMs is that of ceramic coatings, where there is a need to reduce the stress concentrations and to increase the fracture strength [11,12,13,14].

W/Cu FGMs represent a class of materials which combine the properties of W such as high melting point, low coefficient of thermal expansion, and high mechanical resistance with those of copper, namely high electrical and thermal conductivity. Due to these properties, W/Cu FGMs are suitable for plasma-facing materials (PFM) for fusion reactors [15,16,17].

In the literature, there are some processes presented that are used for fabricating W/Cu FGMs, as follows: one-step resistance sintering method [17], laser sintering, vacuum plasma spraying (VPS) [14], chemical vapor deposition (CVD) [18,19], microwave sintering [20], and spark plasma sintering [21,22,23].

Within the methods used by the PM technique, plasma sintering (SPS) is one of the modern processes, which allows for the compaction and sintering of powders at the same time. It is a modern process with great potential [23,24], yet is rarely used in the manufacturing of W/Cu FGMs.

In ref. [25], experimental investigations on the Synthesis of W–Cu Nanocomposite through Spark Plasma Sintering were studied.

In ref. [26], elaboration and thermomechanical characterization of W/Cu Functionally Graded Materials produced by Spark Plasma Sintering for plasma-facing components are presented.

This study is focused on the W/Cu FGMs synthetized by SPS and CS, which were used as starting materials and mechanically milled powders, respectively.

## 2. Materials and Experimental Methods

### 2.1. Material Selections

In order to prepare W/Cu FGM, tungsten nanopowders (44 nm) and copper micron powders (type SE Pometon) were used. The particle size distribution of W nanopowders was determined by Dynamic Laser scattering (DLS), using a 90 Plus particle size analyzer, Brookhaven Instruments Corporation, Holtsville, NY, USA, equipped with 35 mW solid state laser, having 660 nm wavelength. The powder was in suspension with distilled water and the suspension was subjected to an ultrasound process for 5 min. Compared to SEM analysis, DLS measures the hydrodynamic diameter, which can be different because it depends on several factors such as the concentration of the dispersion medium, the type of ions in the dispersion medium, the size of the particles core, and the surface structure of the particles [27,28].

**Figure 1:**
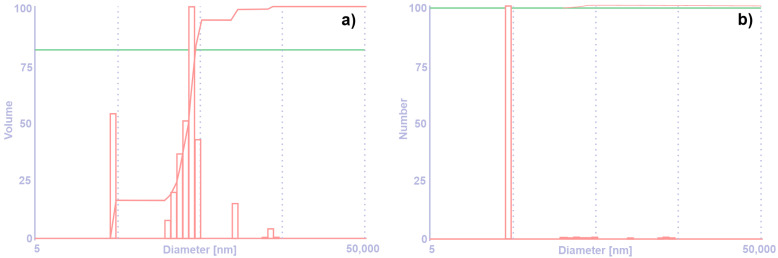
Particle size distribution: (**a**) volume distribution; (**b**) number distribution.

In Figure 1 and Table 1, particle size distributions of W nanopowders are shown.

As it can be seen in Figure 1a, on the volume distribution there are observed more bars which are not visible on the number distribution, Figure 1b. This means that there are particles which are agglomerate, but in a smaller volume. To avoid this phenomenon, it is better to increase the ultrasound time. From Table 1 it was observed that the particle size is equal to 43.7 nm.

**Figure 2:**
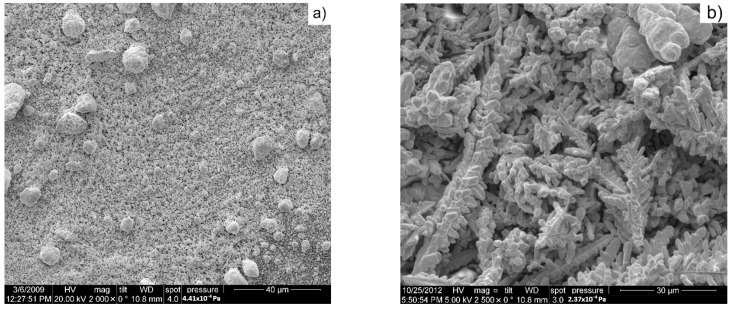
SEM images of initial powders: (**a**) tungsten nanopowders; (**b**) copper powders.

In Figure 2, SEM images of the initial materials are presented. Tungsten nanopowders were obtained by mechanical milling for 35 h and, as it is observed in Figure 2a, these are agglomerated and have classical aspect of milled powders. Indeed, the powders consist of nanostructured particles that create larger particles. Additionally, the larger particles created by the nanopowders coexist with individual nanometric particles.

Copper powders, as seen in Figure 2b, have a dendritical shape due to the electrolytic process that was used in their production. The properties of the Cu powders are presented in Table 2.

Three types of mixtures with the following compositions—(% weight) 80 W/20 Cu, 75 W/25 Cu, and 65 W/35 Cu—were subjected to the mechanical milling (MM) process for 20 h in order to obtain composite powder used for preparing FGMs. For the MM process, a vario-planetary ball mill Pulverisette 4 made by Fritsch was used. The MM parameters were the following: the material of vials was stainless steel, vial volume was 250 mL, the material of the balls was stainless steels, the ball diameter was Φ = 10 mm, the balls to powder ratio was 2/1, the rotational speed of the main disk was 400 rpm, the rotational speed of the vials was 800 rpm, the milling atmosphere was argon, and the milling type was dry.

### 2.2. Experimental Procedure

**Figure 3:**
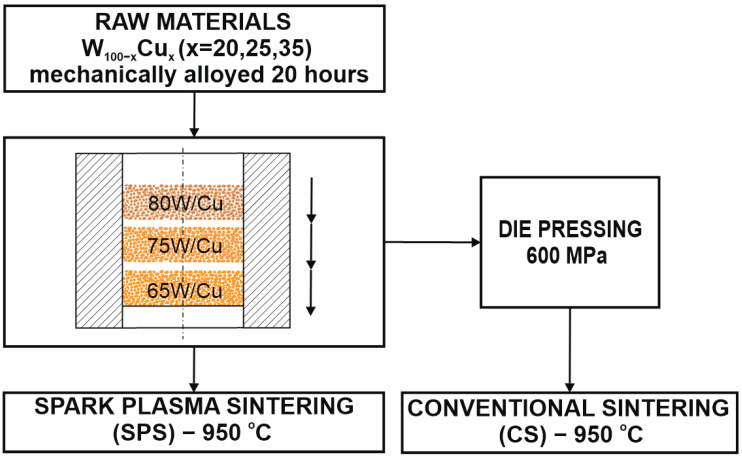
Flow chart of operations for fabricating the FGMs.

The mixtures obtained after MM process were used to fabricate the FGM materials by SPS and CS, according to the flow chart presented in Figure 3.

**Figure 4:**
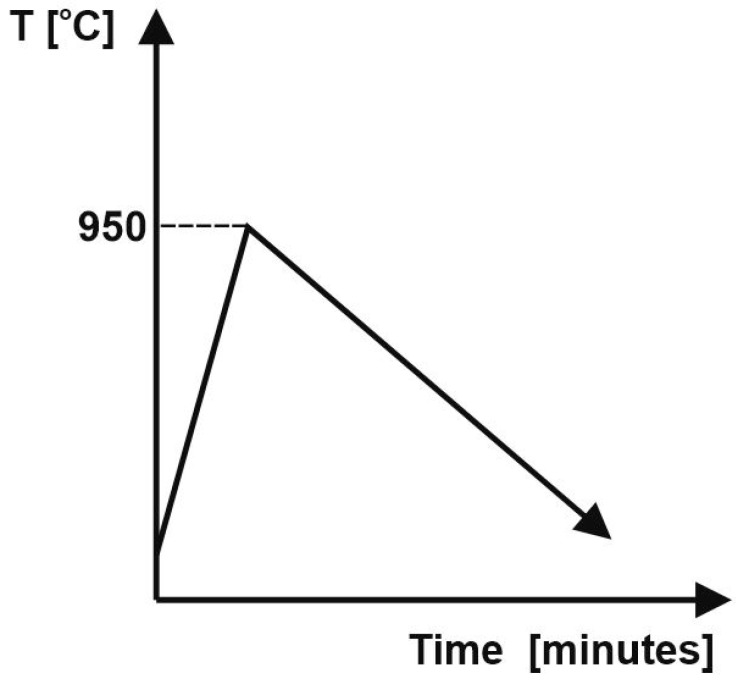
SPS diagram.

For the SPS process the mixtures were stacked layer by layer into a graphite die (Φ = 20 mm) that was cylindrically shaped, according to Figure 3. A thin graphite paper was put between the punches and the powders to prevent the powders from sticking to it and to promote the electrical contact between the punch and powder. The sintering process was carried out according to the diagram given in Figure 4, with the following parameters: final temperature—950 °C; no dwell time; pressure—20 MPa; heating rate—300 °C/min.; current—10,000 A. The sintering parameters were chosen based on the temperature used for CS and it was performed with home-made SPS equipment from the Technical University of Cluj-Napoca. SPS installation operated at 24 V and 3.75 kA. Upon heating, the pulse ON time was 20 ms and the OFF-time was 20 ms.

**Figure 5:**
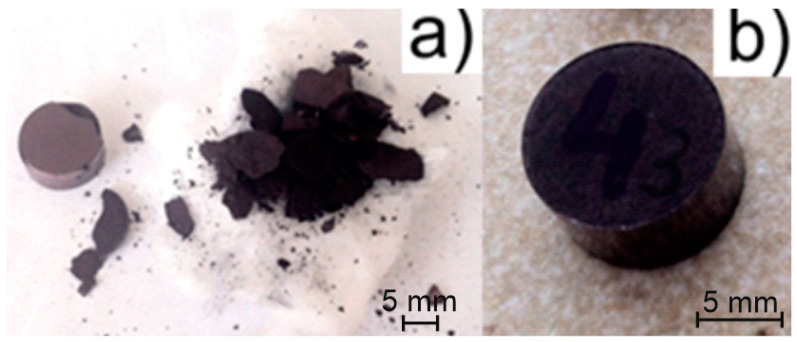
Green W/Cu FGM: (**a**) without binder; (**b**) with 2% binder.

In order to obtain W/Cu FGMs by CS process, the powders were stacked layer by layer into a metallic die (Φ = 12 mm) and were pressed at 600 MPa using LBG testing equipment, which was connected to the computer for registering the results by TCSoft2004Plus software. The force that was applied was equal to 68,293 Kn, the pressing speed was 50 mm/minute, and there was no dwell time at the higher force. In the first experiment, the powders were pressed without any lubricant addition and, because the particles were smaller (less than 500 nm), the green part had no mechanical strength (Figure 5a). Following these results, it was decided that the addition of a binder was necessary. A 2% weight of binder (paraffin) was added, resulting in the part with consistency (Figure 5b). This behavior was expected, as the powder was mechanically processed, and this procedure led to the loss of the powder plasticity, increasing the powder hardening. Additionally, the size of the particles has a great influence in the pressing process, the powders are fine, and the densification process demands a binder.

**Figure 6:**
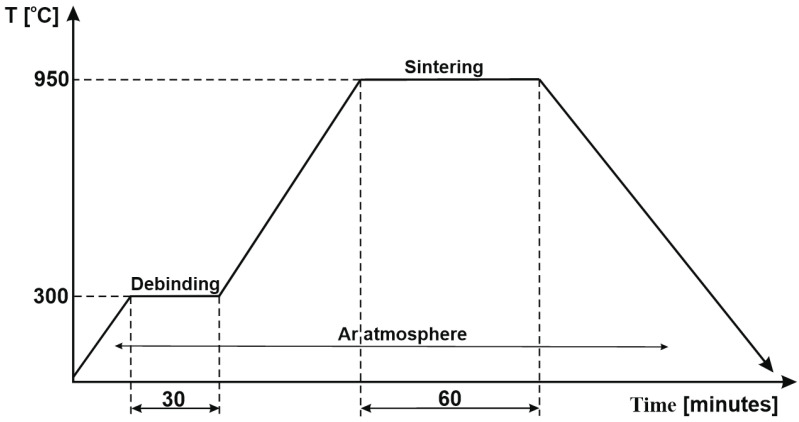
Conventional sintering diagram.

The CS process was carried out according to the diagram given in Figure 6 and consists of a debinding cycle of 30 min. The sintering cycle started at 950 °C with a dwell time equal to 60 min. The heating rate was 10 °C/minute and the cooling was slow, with the furnace (type: Nabertherm L3/11/C6). All cycles were carried out in an argon atmosphere.

The samples were axially cut using a Metkon Metacut-M 250 machine and were heat-mounted in transparent resin (30 mm diameter) using a Metkon Metapress-A machine. In order to study the microstructural aspects, the mounted samples were ground with silicon carbide papers with the following grits: 80, 120, 240, 400, 800, 1200, 2000, and 2500. They were polished with a GALAXY Polishing cloth PHI and Dia-Duo 2 suspension using Metkon Forcipol 2V machine. SEM characterization was performed using a JEOL JSM5600LV microscope equipped with an EDX spectrometer (Oxford Instruments, INCA 200 software).

## 3. Experimental Results and Discussion

**Figure 7:**
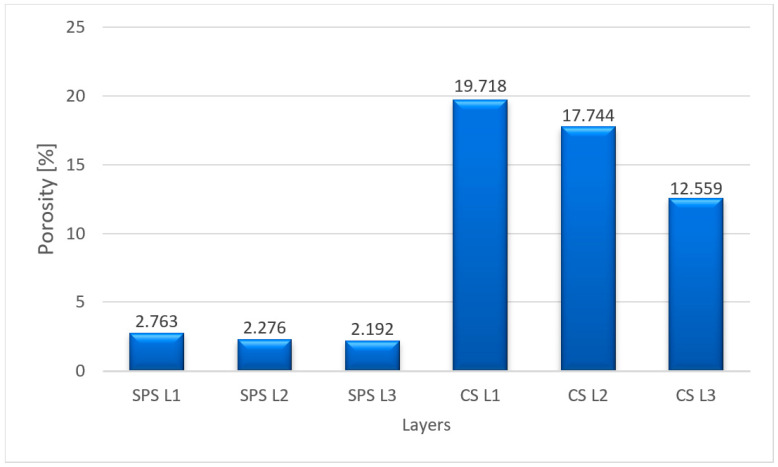
Porosities of the layers.

To determine the densities, the porosities of each layer were measured by image analysis using ImageJ software. In Figure 7, the porosities (P) of each layer for the two processes are presented, such as SPS (SPS L1—layer 1, SPS L2—layer 2, SPS L3—layer 3) and CS (CS L1—layer 1, CS L2—layer 2, CS L3—layer 3).

For each layer, the composition and theoretical density were determined by applying the following Equation:

Dc = DrVr + DmVm(1)

where,

Dc—density of composite material; Dr—density of reinforcement material; Vr—volume fraction of reinforcement material; Dm—density of matrix material; Vm—volume fraction of matrix material.

In order to convert weight percentage to volume percentage, we applied the following equations:

(2) $$V_{A}=\frac{100}{1+\frac{G_{B}\rho_{A}}{G_{A}\rho_{B}}}\;[\%]$$

(3) $$V_{B}=\frac{100}{1+\frac{G_{A}\rho_{B}}{G_{B}\rho_{A}}}\;[\%]$$

where,

*G_A_*—% weight of tungsten; *G_B_*—% weight of copper; $\rho_{A}$—density of tungsten; $\rho_{B}$—density of copper; *V_A_*—% volume of tungsten; *V_B_*—% volume of copper.

Applying the Equations (1)–(3) and considering the density of W and Cu as 19.25 and 8.96 g/cm^3^, the theoretical density ($\rho_{th}$) of each layer was as follows: for layer 1 (80 W/20 Cu)—15.65 [g/cm^3^], for layer 2 (75 W/25 Cu)—14.96 [g/cm^3^], for layer 3 (65 W/35 Cu)—13.73 [g/cm^3^].

The densities were calculated according to the following Equation:

(4) $$\rho =\rho_{th}\left(1-\frac{P}{100}\right)\;\left[\frac{g}{cm^{3}}\right]$$

**Figure 8:**
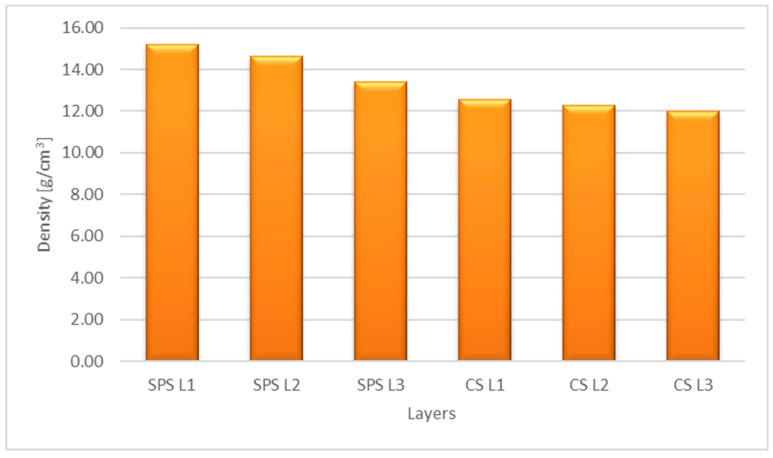
The layers densities after SPS and CS.

In Figure 8, the densities of each layer for the two processes, SPS and CS, are plotted.

As can be seen from Figure 7, a much lower porosity (average porosity of the three layers—2.41%) was attained for the sample sintered by the SPS process compared with the sample obtained by the CS process (average porosity of the three layers—16.67%). Being complementary with porosity, the density is higher for the sample obtained by the SPS process (Figure 8).

**Figure 9:**
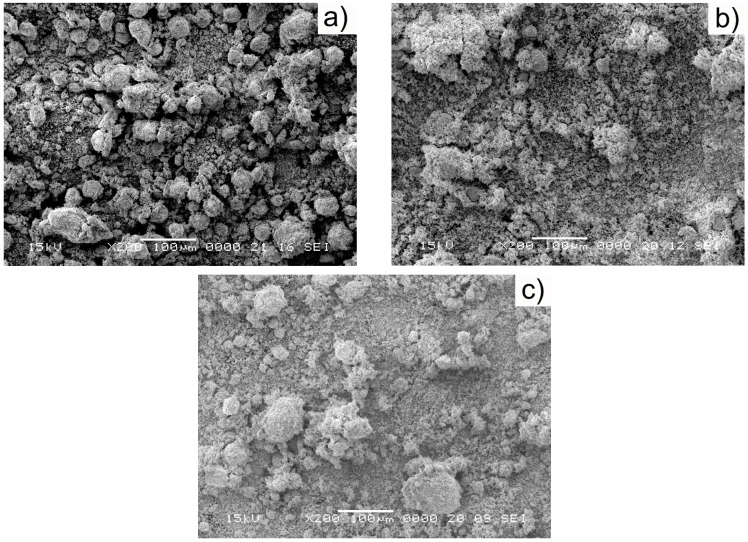
SEM images of the mechanically alloyed powders obtained after 20 h of MA: (**a**) 80 W/20 Cu; (**b**) 75 W/25 Cu; (**c**) 65 W/35 Cu.

After milling together for 20 h, the morphologies of the three W/Cu compositions are shown in Figure 9.

As can be seen from Figure 9, the particles were homogenously dispersed for all the three mixtures. A good mixing of the components was achieved.

**Figure 10:**
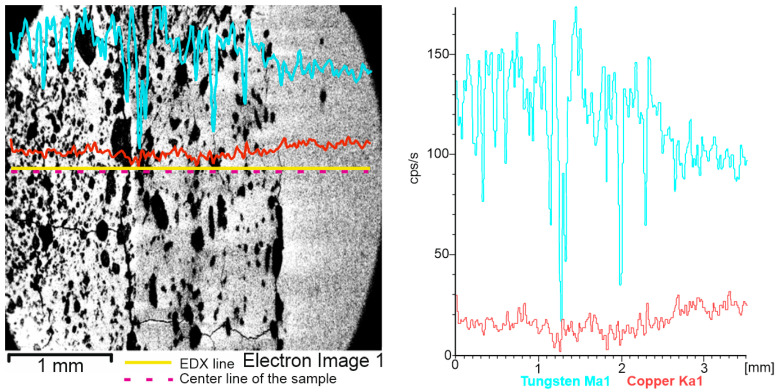
EDX line distribution of W and Cu elements of the W/Cu FGM obtained by CS.

Figure 10 describes the line distribution of the cross-section W/Cu FGM, obtained by CS process. Tungsten content decreased from the first layer (left) to the third layer (right). Due to the porosity of the sample, the distribution of the hard component was affected by the material pores. On other hand, Cu acts as a binder and covers particles of W, leading to a smother distribution on the analyzed line.

**Figure 11:**
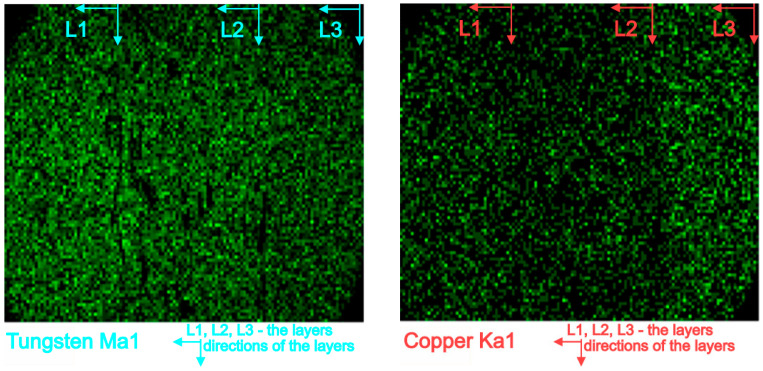
EDX map distribution of W and Cu elements of the W/Cu FGM obtained by CS.

**Figure 12:**
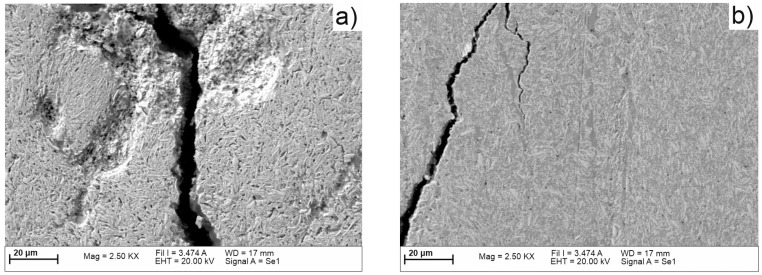
SEM images of the interfaces of W/Cu FGM obtained by CS: (**a**) between layer 1 and layer 2; (**b**) between layer 2 and layer 3.

Figure 11 shows the map’s distribution of the W and Cu elements of the cross-section of the W/Cu FGM obtained by CS, and Figure 12 shows the interface image of the three layers.

The distribution maps of W/Cu FGM obtained by CS, Figure 11, show some discontinuities due to the presence of cracks in the structure of each layer and interface, as can be seen in Figure 10 and Figure 12. The sample has large cracks and can be suitable for practical use if this structure, and other compaction methods, are suitable [29].

**Figure 13:**
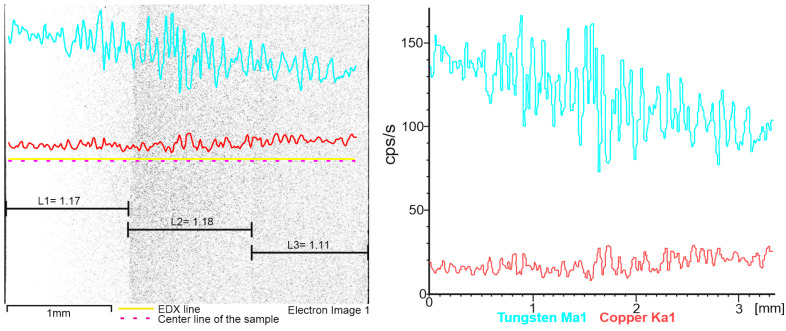
EDX line distribution of W and Cu elements of the W/Cu FGM obtained by SPS.

**Figure 14:**
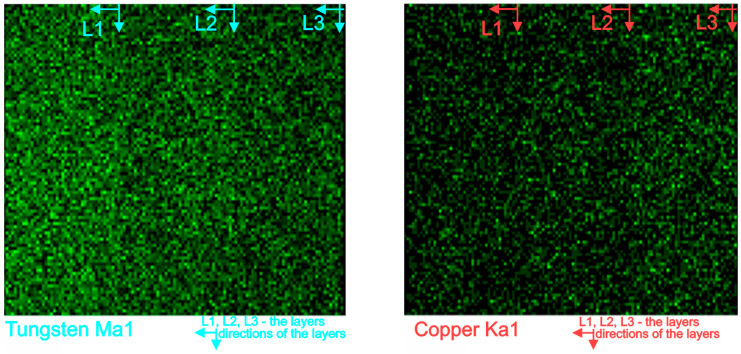
EDX elemental map distribution of W and Cu elements of the W/Cu FGM obtained by SPS.

**Figure 15:**
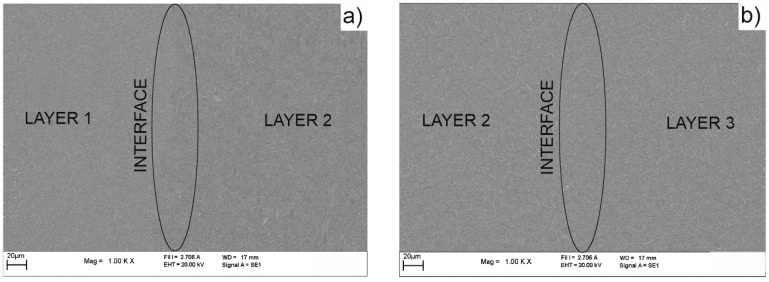
SEM images of the interfaces of W/Cu FGM obtained by SPS: (**a**) between layer 1 (80 W/Cu) and layer 2 (75 W/Cu); (**b**) between layer 2 and layer 3 (65 W/Cu).

In the case of SPS sample, the EDX line distribution, as can be seen from Figure 13, shows the elemental content evolution of tungsten (W) and copper (Cu) as a function of the scanning distance. It was observed that the content of W decreases from the left (first layer—80%W) to the right (third layer—65%W). In Figure 14 the EDX map distributions for the cross-section of the three-layered W/Cu FGM are presented, and in Figure 15 the SEM images of interfaces obtained with the three layers are presented.

According to the maps, it was observed that the W and Cu phase are distributed relatively uniformly in each layer. Analyzing Figure 13 and Figure 14 and the compositions of each layer, it was concluded that the graded structure was attained, having the following dimensions of the layers: layer 1 = 1.17 mm, layer 2 = 1.18 mm, and layer 3 = 1.11 mm. The interfaces seem to present continuity from one layer to another. At a higher magnitude (over 1000×) there are no observed differences in the interface area, as can be seen in Figure 15.

Analyzing the distribution of elements and the images of the interfaces, it can be concluded that, using the SPS method, compact W/Cu FGM materials are obtained.

## 4. Conclusions

Functional graded materials based on W/Cu were successfully fabricated by the SPS process. To obtain green W/Cu FGMs by the CS method it is necessary to add binder, otherwise the green part of the sample has no mechanical strength. The elemental distribution maps of the samples obtained by SPS are more homogenous compared with the samples obtained by the CS process. In the cross-section of the CS samples, the presence of some dimples and cracks was observed, which are due to the pressing process. The cracks are presented in each layer, and in the interfaces. The CS process is time-consuming (~125 min until sintering temperature) compared to SPS sintering (~3 min). In contrast, through SPS a uniform and crack-free sample was obtained. It had lower porosity and high density. According to the morphological analysis, the SPS process is more suitable to obtain W/Cu FGMs compared with the CS process in the case of powders with fine granulation that are used as raw materials.

## Figures and Tables

**Table 1:** Data of the particle size distribution.

d (nm)	G (d)	C (d)	d (nm)	G (d)	C (d)	d (nm)	G (d)	C (d)
31.1	0	0	201.5	0	99	1305.7	0	100
36.8	0	0	238.8	0	99	1547.5	0	100
43.7	100	99	283	0	99	1834.1	0	100
51.8	0	99	335.4	0	100	2173.7	0	100
61.3	0	99	397.5	0	100	2715.8	0	100
72.7	0	99	471.1	0	100	3084.8	0	100
86.2	0	99	558.4	0	100	3504	0	100
102.1	0	99	661.8	0	100	3980	0	100
121	0	99	784.3	0	100	5083	0	100
143.4	0	99	929.6	0	100	6024.2	0	100
170	0	99	1101.7	0	100	7139.8	0	100

**Table 2:** Properties of copper powder.

Physical Properties		
Properties	Admitted Values	Standard
Apparent density [g/cm^3^]	2.30–2.50	SR EN 23923-1/98
Flow time [sec/50 g]	Max 40	SR ISO 4490:2000
Chemical composition		
Element	Admitted Values	Standard
Cu	Min. 99.7	IL-08-0-94
O_2_	Max 0.15	SREN 24491-4:1994

## Data Availability

Not applicable.
